# Interpreting Discordant Monosomy 3 FISH and Chromosomal Microarray Analysis Results in Uveal Melanoma

**DOI:** 10.3390/diagnostics13050946

**Published:** 2023-03-02

**Authors:** Christopher P. Long, Nicholas Coley, John Thorson, Jonathan H. Lin

**Affiliations:** 1 Department of Ophthalmology, Roski Eye Institute, University of Southern California, 1450 San Pablo St #4400, Los Angeles, CA 90033, USA; 2 Diagnostic Pathology Medical Group, 3301 C St, Suite 200E, Sacramento, CA 95816, USA; 3 Department of Pathology, University of California, 9444 Medical Center Drive 1-200, La Jolla, CA 92037, USA; 4 Department of Pathology, Stanford University, 300 Pasteur Dr., Palo Alto, CA 94304, USA; 5 Department of Ophthalmology, Stanford University, 2452 Watson Ct, Palo Alto, CA 94303, USA; 6 VA Palo Alto Healthcare System, 3801 Miranda Avenue, Palo Alto, CA 94304, USA

**Keywords:** uveal melanoma, monosomy 3, microarray, FISH

## Abstract

Uveal melanoma is the most common primary ocular tumor in adults and causes morbidity through lymphovascular metastasis. The presence of monosomy 3 in uveal melanomas is one of the most important prognostic indicators for metastasis. Two major molecular pathology testing modalities used to assess monosomy 3 are fluorescence in situ hybridization (FISH) and chromosomal microarray analysis (CMA). Here, we report two cases of discordant monosomy 3 test results in uveal melanoma enucleation specimens, performed using these molecular pathology tests. The first case is of uveal melanoma from a 51-year-old male that showed no evidence of monosomy 3 when assessed by CMA, but where it was subsequently detected by FISH. The second case is of uveal melanoma from a 49-year-old male that showed monosomy 3 at the limit of detection when assessed by CMA, but where it was not detected by subsequent FISH analysis. These two cases underscore the potential benefits of each testing modality for monosomy 3. Mainly, while CMA may be more sensitive to low levels of monosomy 3, FISH may be best method for small tumors with high levels of adjacent normal ocular tissue. Our cases suggest that both testing methods should be pursued for uveal melanoma, with a single positive result for either test interpreted as indicating the presence of monosomy 3.

## 1. Materials and Methods

An oncoscan microarray was performed at the University of California San Diego (UCSD) Center for Advanced Laboratory Medicine (CALM), using the Affymetrix/Thermofisher OncoScan platform (catalog number: 902695), on DNA extracted from sections of formalin-fixed, paraffin-embedded (FFPE) tissue. Monosomy 3 FISH was performed at Mayo Clinic Laboratories (test ID: UMM3F), utilizing the D3Z1 centromeric probe (Abbot Molecular) and the BCL6 long-arm probe (Mayo Laboratories). Greater than 28% of 200 cells lacking two chromosome 3 signatures is requisite for a positive monosomy 3 result when using this assay. Next-generation sequencing (NGS) was performed at the UCSD CALM on DNA extracted from FFPE tissue using a laboratory-developed 397 gene hybrid capture-based assay, with analysis conducted on an Illumina HiSeq 2500 instrument (Illumina, San Diego, CA, USA).

The images of FFPE, hematoxylin- and eosin (H&E)-stained whole-eye cross sections were taken using the Aperio AT2 whole-slide scanner. The remaining images were captured using the Olympus SC30 camera with cellSens software, attached to an Olympus BX43 microscope.

The UCSD institutional review board approved the use of the collected tissue samples and associated clinical information for this study. The research was Health Insurance Portability and Accountability Act (HIPAA)-compliant and adhered to the principles of the Declaration of Helsinki.

## 2. Figure and Table Legends

Patient #1 is a 51-year-old male who was referred to ophthalmology for progressive left-sided vision loss. Ophthalmoscopic examination revealed a mushroom-shaped pigmented lesion, emanating from the posterior uvea overlying the optic nerve, which was proven to be melanoma by the performance of a fine-needle aspiration (FNA) biopsy. He underwent a 7-day surgical placement of radioiodine plaque overlying the tumor 1 month after initial pathologic diagnosis. A clinically detected local recurrence at the periphery of the plaque site, detected 60 months after pathologic diagnosis, underwent multiple rounds of laser ablation. The patient ultimately underwent enucleation 79 months after pathologic diagnosis. His most recent imaging, taken 92 months after initial pathologic diagnosis, demonstrates numerous liver metastases and local left orbital tumor extensions, tracking along the optic nerve to involve the optic chiasm.

**Figure 1:**
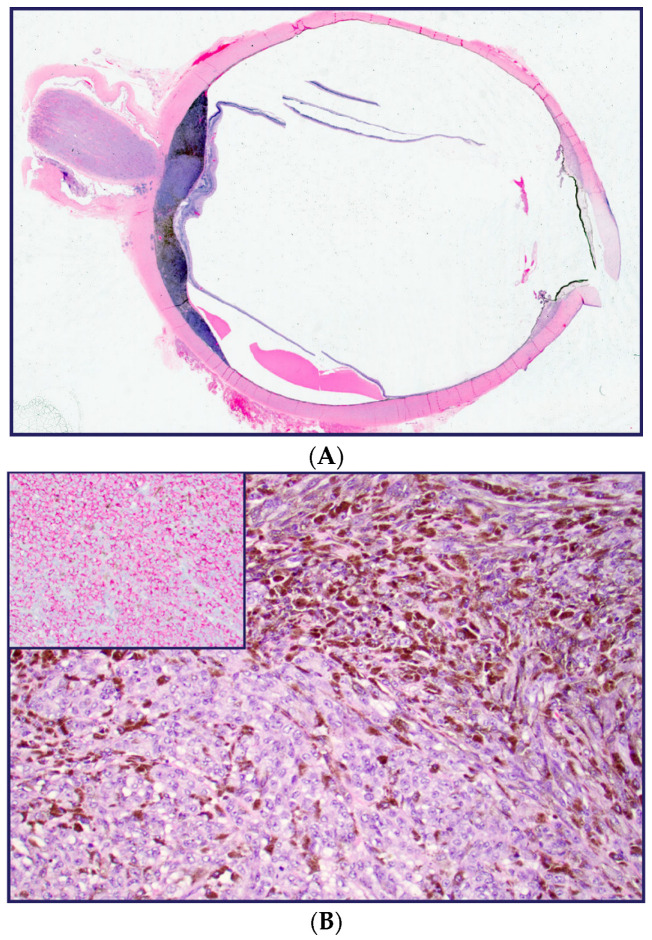
Melanoma from patient #1 without extrascleral extension (**A**). The tumor assumes a primarily epithelioid morphology (**B**), with the expression of HMB45 in malignant melanocytes (insert).

As seen in Figure 1, the melanoma from patient #1 arises from the posterior uvea overlying the optic nerve. A focus on scleral invasion adjacent to the optic nerve is present, but no extrascleral extension is noted (A). The tumor assumes a primarily epithelioid morphology (B), with the expression of HMB45 in malignant melanocytes (insert).

Patient #2 is a 49-year-old male who visited ophthalmology for an enlarging pigmented mass present on his right iris. He underwent surgical radioiodine plaque placement (removed after 7 days) at the time of an FNA biopsy that demonstrated uveal melanoma. He underwent a 7-month trial of sunitinib shortly after initial pathologic diagnosis. Fifteen months after pathologic diagnosis, magnetic resonance imaging (MRI) of the abdomen demonstrated two biopsy-proven metastatic liver lesions. These were subsequently treated with radioablation. He underwent enucleation 36 months after pathologic diagnosis and a right apical lung lesion, biopsied at 38 months, demonstrated metastatic melanoma. An enlarging right apical lung lesion was the only metastatic disease detected by computed tomography (CT) of the chest in the patient’s most recent imaging scan, performed 48 months after pathologic diagnosis.

**Figure 2:**
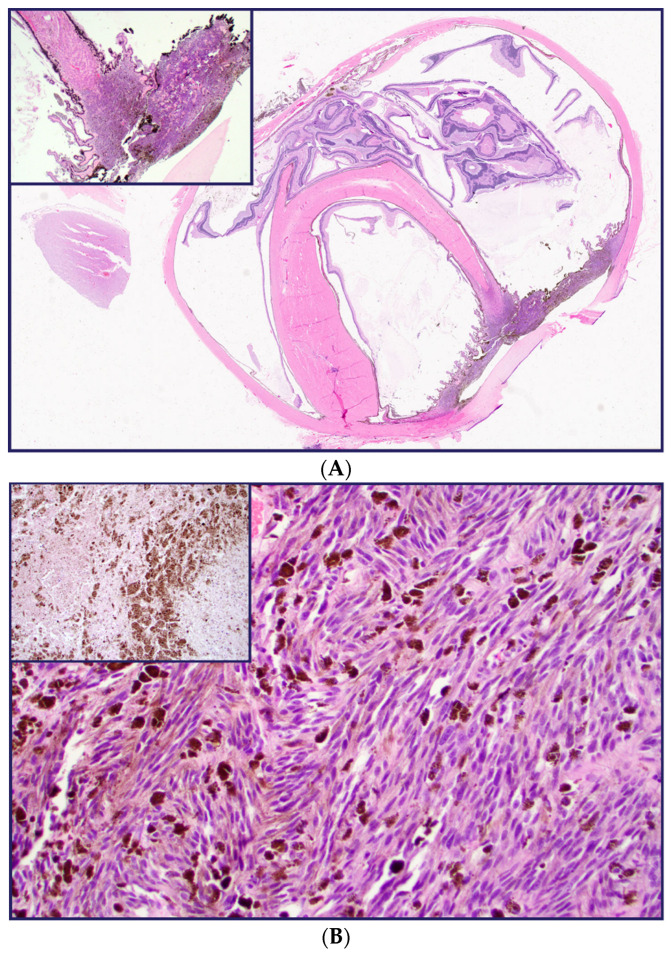
Melanoma from patient #2 (**A**) originating from ciliary body (insert). Tumor sample with spindle-cell morphology (**B**), necrosis, melanophages, secondary to previous treatment (insert).

As demonstrated in Figure 2, the melanoma from patient #2 (A) arises from the ciliary body (Insert). The tumor assumes a primarily spindle-cell morphology (B), characterized by extensive necrosis, and the presence of melanophages, secondary to radioiodine plaque treatment (Insert).

**Figure 3:**
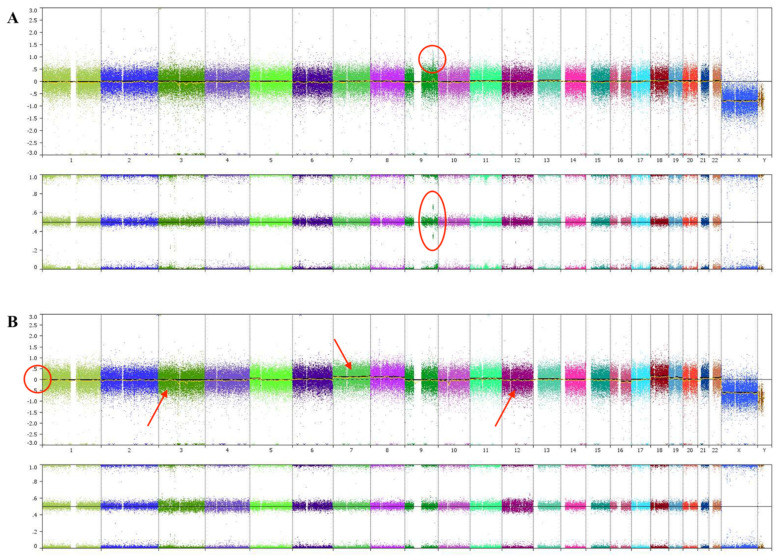
Raw CGH microarray relative fluorescence data for the uveal melanoma from patient #1 (**A**) and patient #2 (**B**).

Figure 3 demonstrates the raw CMA microarray relative fluorescence data for the uveal melanoma from patient #1 (A) and patient #2 (B). For each patient, chromosome position/array probe location is located on the *x* axis of each panel. Relative copy number is denoted on the *y* axis of the top panel of data for both patients (A and B), while B-allele frequency is depicted in the lower panel of data for each patient. The uveal melanoma from patient #1 shows segmental gain of chromosome 9q, as evidenced by (1) the slight spike in the relative copy number plot (circled) and (2) the same region of the B-allele frequency plot showing an allele frequency of approximately 0.4 or 0.6 (also circled). Whereas the normal genotypes of AA, AB, or BB produce the expected B-allele frequencies of 0, 0.5, or 1.0, segmental gains result in an AAB genotype (i.e., gain of a A allele copy), leading to a b-allele frequency of >0 but <0.5 or an ABB genotype (i.e., gain of a B allele copy), producing a B-allele frequency of >0.5 but <1.0. The uveal melanoma from patient #2 shows low frequency loss of chromosome 3, 4, 12, and 16q with gains of 7, 8, 18, 19, and 22. These changes can best be appreciated by inspection of the copy number plot for patient #2 (Figure 3B, top panel; see arrows) where slight shifts of the average copy number above or below the 0 line indicate a loss (movement below the 0 line) or a gain (movement above the 0 line) of chromosomal material.

As summarized in Table 1, no clinically significant sequence variants were detected by NGS for either tumor. The uveal melanoma from patient #1 showed focal gain of chromosome 9 and no detectable chromosomal losses. However, this tumor demonstrated monosomy 3 when examined by FISH analysis. The uveal melanoma from patient #2 showed several chromosomal gains in addition to monosomy 3 at the limit of detection. FISH analysis did not detect monosomy 3 for this tumor.

These two cases highlight the diagnostic strengths and pitfalls of CMA and FISH for assessing monosomy 3 status in uveal melanoma [1,2,3,4,5]. CMA utilizes DNA extracted from an entire tumor sample and inevitably includes some background or interspersed normal tissues [6]. Consequently, CMA may fail to detect monosomy 3 in a small subclone or the DNA from normal tissue may obfuscate low-level loss of chromosome 3 within a tumor [6]. The detection of monosomy 3 by FISH, while it was not detected by CMA in patient #1, may reflect this phenomenon. FISH, however, samples a narrow plane of tissue and requires a relatively high proportion of cells with a single probe signal to minimize false positive results [6,7]. Thus, FISH may miss monosomy 3 at a low level that CMA can routinely detect [6,7]. The detection of monosomy 3 by CMA, a substance not detected by FISH in patient #2, may reflect this phenomenon. Overall, these two cases highlight the strengths and weaknesses of FISH and CMA as assays to detect monosomy 3 in uveal melanoma. We suggest that both FISH and CMA should be performed for uveal melanoma, with a positive result for either test interpreted as indicating the presence of monosomy 3.

## Figures and Tables

**Table 1:** Clinical Summary of CMA and FISH results of patient #1 and patient #2.

	Patient #1	Patient #2
Mutations	No clinically significant variance detected.	No clinically significant variance detected.
Chromosomal Gains	2.27 Mb gain in 9q33.1 encompassing 6 genes (ASTN2, SNORA70C, LOC101928797, TLR4, LINC02578, BRINP1).	Gain of chromosomes 7, 8, 18, 19, and 22.
Chromosomal Losses	No chromosomal losses detected.	Monosomy 3 at limit of detection with additional losses of chromosomes 4, 12, and 16q.
Monosomy 3 FISH Status	Positive, 63.5% of 200 cells counted (28% cutoff).	Negative.

## Data Availability

FFPE tissue from either case can be obtained for additional studies following approval of a material transfer agreement. Microarray data from each case can be provided de-identified of HIPAA sensitive information upon request as well as the original FISH reports from Mayo laboratories.
